# Placing joy, surprise and sadness in space: a cross-linguistic study

**DOI:** 10.1007/s00426-016-0787-9

**Published:** 2016-07-18

**Authors:** Fernando Marmolejo-Ramos, Juan C. Correa, Gopal Sakarkar, Giang Ngo, Susana Ruiz-Fernández, Natalie Butcher, Yuki Yamada

**Affiliations:** 1 Gösta Ekman Laboratory, Department of Psychology, Stockholm University, Frescati Hagväg 9A, 106 91 Stockholm, Sweden; 2 Fundación Universitaria Konrad Lorenz, Bogotá, Colombia; 3 Department of Computer Applications, G.H. Raisoni College of Engineering, Nagpur, India; 4 School of Education, The University of Adelaide, Adelaide, Australia; 5 Leibniz Knowledge Media Research Center, Tübingen, Germany; 6 School of Social Sciences, Business and Law, Teesside University, Middlesbrough, UK; 7 Faculty of Arts and Science, Kyushu University, Fukuoka, Japan

## Abstract

The valence–space metaphor posits that emotion concepts map onto vertical space such that positive concepts are in upper locations and negative in lower locations. Whilst previous studies have demonstrated this pattern for positive and negative emotions e.g. ‘*joy*’ and ‘*sadness*’, the spatial location of neutral emotions, e.g. ‘*surprise*’, has not been investigated, and little is known about the effect of linguistic background. In this study, we first characterised the emotions *joy*, *surprise* and *sadness* via ratings of their concreteness, imageability, context availability and valence before examining the allocation of these emotions in vertical space. Participants from six linguistic groups completed either a rating task used to characterise the emotions or a word allocation task to implicitly assess where these emotions are positioned in vertical space. Our findings suggest that, across languages, gender, handedness, and ages, positive emotions are located in upper spatial locations and negative emotions in lower spatial locations. In addition, we found that the neutral emotional valence of *surprise* is reflected in this emotion being mapped mid-way between upper and lower locations onto the vertical plane. This novel finding indicates that the location of a concept on the vertical plane mimics the concept’s degree of emotional valence.

## Introduction

Interdisciplinary evidence from robotics (Marocco, Cangelosi, Fischer, & Belpaeme, 2010), neuroscience (Hauk, & Pulvermüller, 2011) and cognitive psychology (Bekkering, & Neggers, 2002) support the so-called theory of embodied cognition (Barsalou, 2008). This theory argues that the processing of concepts is associated with the activation of perceptual and motor systems (see Barsalou, 2008; Binder, & Desai, 2011), and such an association is bidirectional, i.e. the activation of sensorimotor systems affects conceptual processing (e.g. see experiments in Rueschemeyer, Lindemann, van Rooj, van Dam, & Bekkering, 2010), and the activation of concepts affects sensorimotor systems (e.g. see experiment in Glenberg, & Kaschak, 2002). The relationship between concepts and sensorimotor systems is considered essential for effective social cognition, a type of cognition used in everyday life situations.^1^ That is, for example, our perceptual and motor system can influence our cognitive processes (e.g. judgment, thinking, decision-making), just as these processes can influence our physical actions in social contexts (e.g. Wilson, 2002).

Based on this theory, Casasanto (2009) proposed the body-specificity hypothesis (BSH). The BSH argues that people implicitly associate positive-valenced concepts with the side of their bodily space on which they are more skilful. The experiments by Casasanto (2009) supported this prediction showing that right-handers were more likely than left-handers to associate the right space with positive ideas and the left space with negative ideas, whilst the opposite holds true for left-handed participants. Accordingly, right- and left-handers tended to link good things such as intelligence, attractiveness, honesty, and happiness more strongly with their dominant side. In employing functional magnetic resonance imaging (fMRI) to compare right- and left-handers’ brain activity during motor imagery tasks and action verb understanding, Casasanto (2011) found that whilst left-hemisphere motor areas were activated in right-handers, right-hemisphere motor areas were activated in left-handers. This finding lends additional support to the BSH from a neuroscience perspective.

In addition to this, Ansorge and Bohner (2013; see also Ansorge, Khalid, & König, 2013) reported a congruency effect when subjects had to categorise spatial words like *up* as elevated or less elevated (i.e. as high or low in the vertical space), as well as categorise affective words like *happy* as positive or negative. Their results support the assumption that valence–vertical space associations exist in semantic memory, so that faster responses were observed when target words were presented in spatially congruent locations (e.g. *happy* in the upper part of a computer screen). Similarly, Meier and Robinson (2004) found that positive-valenced words activated higher areas of visual space, whilst negative words activated lower areas of visual space (Study 2; see also Xie, Wang, & Chang, 2014), and Sasaki, Yamada and Miura (2015) showed that the emotional valence of images is influenced by motor action towards the upper or lower vertical spatial location (see also Sasaki, Yamada, & Miura, 2016).

**Fig. 1:**
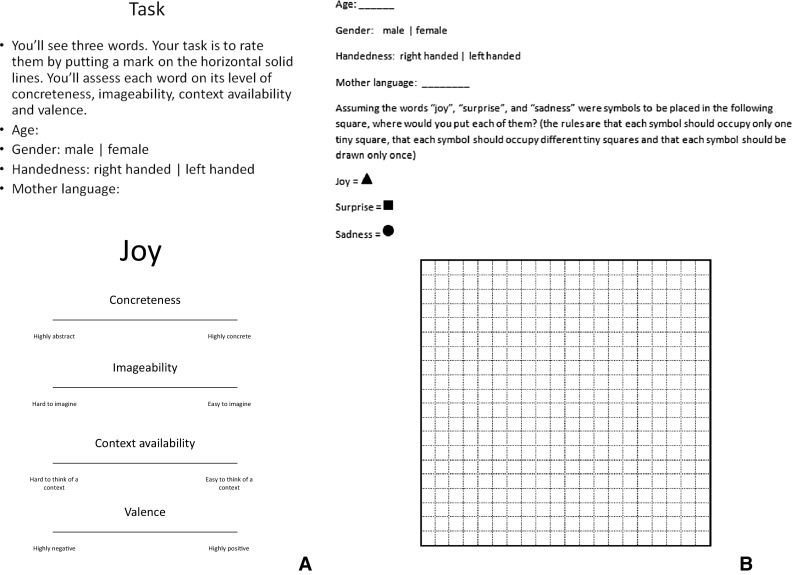
Materials used in the rating (**a**) and the word allocation (**b**) tasks. **a** The case of joy for illustrative purposes only

To further expand on these previous studies, Marmolejo-Ramos, Elosúa, Yamada, Hamm, and Noguchi (2013) examined whether a dominance of the vertical plane exists over the horizontal plane. Their results supported the predictions of the BSH described above, but also showed that the vertical plane is more salient than the horizontal plane in relation to the allocation of valenced words. That is, whilst a rating task showed that left-handers rated the word *left* as more positive than *right* and right-handers showed the opposite pattern, a word allocation task showed that positively valenced words were placed in upper locations, whereas negatively valenced words were placed in lower locations regardless of participants’ handedness. Thus, the results lend support to the BSH and also indicate a higher saliency of the vertical plane over the horizontal in the allocation of valenced words (recent evidence as to the saliency of the vertical plane over the horizontal plane is further reported by Damjanovic, & Santiago, 2016). Note that Marmolejo-Ramos et al. (2013) reported some differences in the rating task amongst several linguistic groups (see Fig. 1 in their paper), but there were no linguistic differences in the word allocation task.

However, in a recent specialised section devoted to research in embodied cognition (Marmolejo-Ramos, & D’Angiulli, 2014), one article reported a study about the effect of linguistic factors on the valence–space metaphor. Marmolejo-Ramos, Montoro, Elosúa, Contreras, and Jiménez-Jiménez (2014) evaluated whether gender and cultural factors have an effect on the mapping of valenced sentences on the vertical space. In the first experiment, Colombian and Spaniards had to recall and report specific personal situations or contexts related to *joy, sadness, surprise, anger, fear,* and *disgust;* i.e. participants recalled and reported situations or contexts in which these emotions occur. Results showed that females expressed more contexts than males, and importantly, Colombians reported more contexts than Spaniards. Based on these results, the researchers designed a new spatial–emotional congruency verification task including sentences that recreated the most representative contexts for the emotions of *joy* and *sadness* (e.g. John had a good time with his friends). After reading a sentence, participants had to judge whether a probe word, displayed in either a high or low position on the screen, was congruent or incongruent with the previous sentence. The results showed a mapping between emotions and vertical space induced by sentences recreating representative emotional contexts. This evidence is in line with research (e.g. Schubert, 2005) suggesting that perceptions and judgments of abstract concepts are processed in metaphorical ways by estimating their relative position inside a vertical space.

The emotion words *joy* and *sadness* are exemplars of positive and negative emotions that have been studied in the context of other valenced concepts (see for an example, the classic study by Bradley and Lang, 1999). Whilst the words *joy* and *sadness* represent highly positive- and highly negative-valenced concepts that are readily mapped onto upper and lower locations in space (e.g. Ansorge, & Bohner, 2013), it is unknown how emotion words with rather neutral valence would be mapped onto space. An emotion word that seems to have a rather neutral valence (e.g. Reali, & Arciniegas, 2015) and whose metaphorical location onto space has not been investigated is that of *surprise. Surprise* is broadly defined as the detection of unexpected situations that challenge a person’s beliefs (Reisenzein, 2009, Reisenzein, Meyer, & Niepel, 2012). It is a peculiar emotion that seems to swing between being negative (e.g. when a person is victim of a robbery) and also positive (e.g. when a person finds his friends at home to celebrate his birthday; see also Macedo, Cardoso, Reisenzein, Lorini, & Castelfranchi, 2009). Also, it has been found that less verbal contexts can be reported for *surprise* compared to emotions such as *joy* and *sadness* (Marmolejo-Ramos et al., 2014). Interestingly, though this emotion has not been studied in the context of embodiment, therefore, the current study aims to do so along with the previously examined emotions; joy and sadness.

The first step before investigating how these three emotions are mapped onto space is to characterise them regarding their level of concreteness (i.e. the degree to which the concept denoted by a word refers to a perceptible entity (Brysbaert, Warriner, & Kuperman, 2014)], imageability [i.e. the ease with which a word gives rise to a sensory mental image of the word (Paivio, Yuille, & Madigan, 1968)], context availability [i.e. the ease with which a context can be brought to mind in which the person would feel that emotion (Schwanenflugel, & Shoben, 1983)] and valence [i.e. the level of positive–negative emotional state attached to what the emotion concept refers to (see Grühn, & Scheibe, 2008)]. The first objective of the study was met by having several linguistic groups rate these three emotion words. Having the ratings from several linguistic groups enables us to gain a comprehensive picture of these emotion words with regards to the levels listed above. Although linguistic differences are expected in the rating of words (see Fig. 1 in Marmolejo-Ramos et al., 2013), it is hypothesised that, across linguistic groups, these emotions could have medium-to-low levels of concreteness, and medium-to-high levels of imageability and context availability. As shown in Table 1, such levels are expected based on previous studies in which the average concreteness, imageability and context availability ratings for the words joy, surprise and sadness have been reported (see Altarriba, Bauer, & Benvenuto, 1999; Altarriba, & Bauer, 2004; Brysbaert et al., 2014).^2^

**Table 1:** Mean concreteness, imageability, context availability and valence ratings of three emotion words as reported in previous studies

Emotion word	Concreteness	Mean rating imageability	Context availability	Valence
Joy	2.37	3.7	5.2	8.60
Surprise	3.24	4.2	4.9	7.47
Sadness	1.82	4.0	5.1	1.61

Altarriba and colleagues (Altarriba et al., 1999; Altarriba, & Bauer, 2004) and Bradley and Lang (1999), used the words ‘surprised’ instead of ‘surprise’ and ‘sad’ instead of ‘sadness’. Brysbaert et al. (2014) provided ratings for ‘joy’, ‘surprise’, ‘surprised’, ‘sad’ and ‘sadness’. The concreteness ratings were performed on a five-point Likert scale and were reported in Brysbaert et al (2014) (note that the concreteness ratings for the words ‘joy’, ‘surprise’ and ‘sadness’ reported by Altarriba and colleagues were 3, 3, and 3.1, respectively, on a seven-point Likert scale). The imageability and context availability ratings were performed on a seven-point Likert scale and were reported in Altarriba et al. (1999). The valence ratings were performed on a nine-point Likert scale and were reported in Bradley and Lang (1999)

In regard to *surprise*, it most likely exhibits lower context availability than *joy* (and possibly *sadness*) as found by Marmolejo-Ramos et al. (2014; see Tables 1, 2 in the article). Note that, in that study, participants generated verbal contexts representing six different emotions, including the three emotions studied herein. These researchers found that *surprise* had the lowest number of verbal contexts (*joy* had the highest number of verbal contexts, followed by *fear* and *sadness*). Thus, it is expected to support such finding via a rating task. It could be speculated that fewer verbal contexts and lower context availability ratings for the concept of *surprise* could be attributed to the neutrality of the concept, which, in turn, may hinder thinking of clear-cut scenarios associated with that given emotion.

**Table 2:** Demographic and descriptive statistic information of the participants in Study 1 and 2 (MAD = median absolute deviation)

Language	Handedness and gender				Total	Age	
	Right-handed		Left-handed			Range	Median (MAD)
	Male	Female	Male	Female			
+++Study 1 (rating task)							
English	5	36	1	8	50	19–54	20 (1.48)
Hindi	20	23	1	1	45	18–26	22 (1.48)
Japanese	4S	40	5	2	95	18–21	19 (0)
Spanish	22	7	2	C	31	18–26	20 (1.48)
Vietnamese	3	34	15	2	54	17–27	19 (0)
German	17	24	4	5	50	19–37	23 (1.48)
Total	115	164	28	18	325		
Total (handedness)	Right-handers = 279		Left-handers = 46				
Total (gender)	Males = 143		Females = 182				
Total age range						17–54	
Total average age (MAD)							20 (1.48)
+++Study 2 (word allocation task)							
English	10	38	1	2	51	19–48	20 (1.48)
Hindi	22	24	1	1	48	18–26	22 (1.48)
Japanese	82	33	5	3	123	18–23	19 (1.48)
Spanish	11	18	2	2	33	18–60	24 (7.41)
Vietnamese	4	37	14	2	57	17–27	19 (0)
German	10	28	5	7	50	18–45	24.5 (4.44)
Total	139	178	28	17	362		
Total (handedness)	Right-handers = 317		Left-handers = 45				
Total (gender)	Males = 167		Females = 195				
Total age range						17–60	
Total average age (MAD)							20 (1.48)

The data were obtained in the following institutions: Teesside University (UK), G.H. Raisoni College of Engineering (India), Kyushu University (Japan), Universidad Simon Bolivar (Venezuela), Hanoi University (Vietnam), and Leibniz Knowledge Media Research Center (Germany)

Regarding emotional valence, it is expected that *joy* will be rated as highly positive, whilst *sadness* will be rated as highly negative. This result has also been reported in previous studies (see Table 1). In the ratings reported in Bradley and Lang (1999), *surprise* seems to lean towards positivity (see Table 1). However, based on theoretical accounts arguing that *surprise* is a rather neutral emotion (e.g. Macedo et al., 2009), we expect that the valence ratings will indicate that surprise is, in fact, neutral.

With regard to the levels of concreteness, context availability, imageability and valence of each emotion word, some variability due to linguistic differences can be expected (see Evans, & Levinson, 2009). This will ultimately be reflected in language effects in all of the 12 rating conditions [i.e. three emotion words (*joy*, *surprise*, and *sadness*) × four word rating dimensions (concreteness, context availability, imageability, and valence)].

The second objective of the study was to investigate the allocation of these three emotions in space via various linguistic groups. Finding that the positive emotion *joy* and the negative emotion *sadness* are placed on upper and lower spatial locations, respectively, would support the findings of Ansorge and Bohner (2013; see also Ansorge et al., 2013; Meier, & Robinson, 2004; Xie et al., 2014, 2015). Indeed, finding that right-handers place the words *joy* and *sadness* towards rightward and leftward spatial locations, respectively, would lend extra support to the BSH (see Casasanto, 2009, 2011). However, based on the results by Marmolejo-Ramos et al. (2013), the distance between *joy* and *sadness* on the horizontal plane (i.e. BSH) is expected to not be significant; rather, it is hypothesised a significant difference between *joy* and *sadness* on the vertical plane exclusively.^3^ These findings would then lend support to evidence suggesting a saliency of the vertical plane over the horizontal plane (see Fig. 2f in Marmolejo-Ramos et al., 2013). Finding that *surprise* is located half-way between the vertical locations of *joy* and *sadness* would show for the first time that *surprise*’s emotional valence is mapped onto space. Specifically, we expect to find that given the neutral valence of *surprise*, this word would be mapped onto a vertical location near the mid-point (i.e. placed between *joy* and *sadness*). The non-linguistic differences originally reported by Marmolejo-Ramos et al. (2013) in the allocation of valenced words onto space suggest that there could be minimal chances of finding language effects in the allocation of these words.

**Fig. 2:**
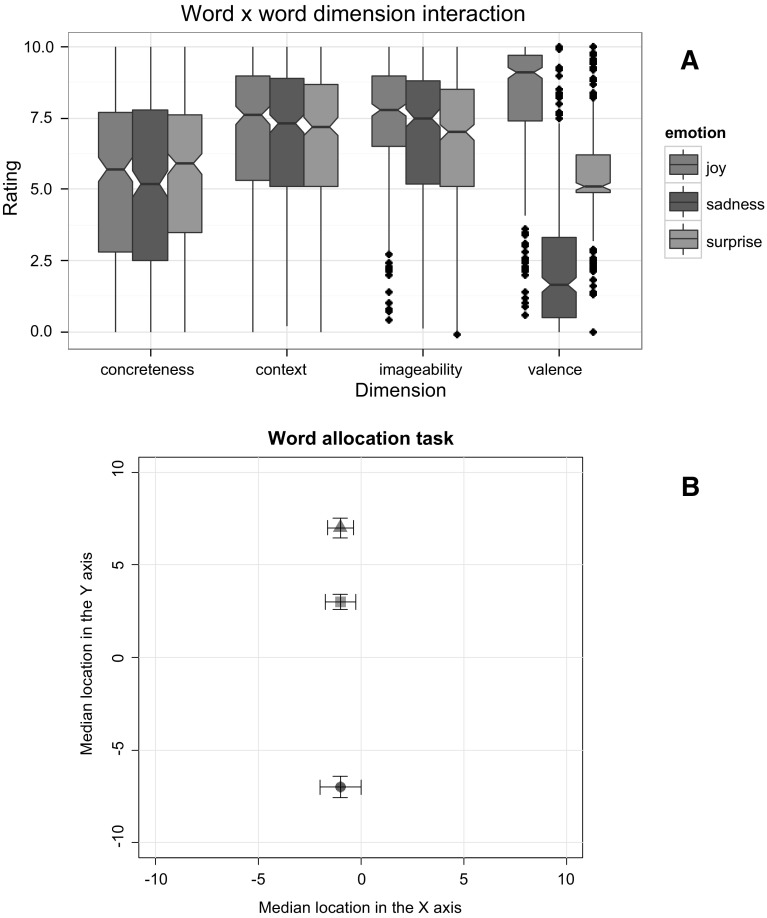
Results of the rating (**a**) and the word allocation (**b**) tasks. The notches in the *box plots* and the *error bars* represent 95 % CI around the median. *Closed triangle* = joy, *closed square* = surprise and *closed circle* = sadness

## Methods

### Participants

University undergraduate students and members of the community from six different linguistic backgrounds (i.e. English, Hindi, Japanese, Spanish, Vietnamese and German) voluntarily participated in the rating (*n* = 325) and the word allocation (*n* = 362) tasks. The experimental protocol was approved by the ethics committees of the institutions involved in the studies. Participants gave written informed consent to abide by the principles of the Declaration of Helsinki. Table 2 reports demographic and descriptive statistic information of the participants (participants whose responses reflected a lack of understanding of the instructions were illegible, or were incomplete and were discarded. Also, participants with incomplete demographic data, e.g. no information about gender, handedness, age or language, were not included in the analyses).

## Materials

The three emotion words *joy*, *surprise* and *sadness* were used in the rating study. The ratings were performed via a simple paper-based task (see Fig. 1a). The word allocation task also consisted of a paper-based task (see Fig. 1b).

### Procedure

#### Rating task

Participants were asked to rate the three emotions on the following dimensions: concreteness, imageability, context availability and valence. The ratings were made by placing a mark (e.g. via a pen or a pencil) on 10-cm horizontal lines; one line for each attribute. On the left end, the scales were labelled as ‘highly abstract’ (concreteness scale), ‘hard to imagine’ (imageability scale), ‘hard to think of a context’ (context availability scale) and ‘highly negative’ (valence scale). On the right end, the scales were labelled as ‘highly concrete’ (concreteness scale), ‘easy to imagine’ (imageability scale), ‘easy to think of a context’ (context availability scale) and ‘highly positive’ (valence scale). The three words were presented to participants for rating in a random order; however, the order of each rating (concreteness, imageability, context availability and valence) for each word was given in a fixed order (see Fig. 1a).

#### Word allocation task

Participants were asked to locate three symbols representing the words *joy*, *surprise* and *sadness* on a 10-cm^2^ gridded square (this grid resembles that used in Experiment 2 by Marmolejo-Ramos et al., 2013). A triangle represented *joy*, a square represented *surprise* and a circle represented *sadness*, and this matching was used for all participants (see Appendix for supplementary results that reflect the counterbalanced emotion/symbol combinations). The instructions read: “assuming the words *joy*, *surprise* and *sadness* were symbols to be placed in the following square, where would you put them?” Participants were also instructed that each symbol should occupy only one square within the grid, each symbol should occupy different squares in the grid, and each symbol should be drawn only once (see Fig. 1b). There were no time restrictions to complete this task.

### Design and analyses

The data in both tasks were analysed via high-breakdown and high-efficiency robust linear regression modelling (see Yohai, 1987) via the ‘lmRob’ function in the ‘robust’ R package. For the rating study, the independent variables were participant, i.e. all participants in rating study (P), language, i.e. the six languages studied (L), gender, i.e. males and females (G), handedness, i.e. right- and left-handers (H), age, i.e. the ages of the participants in the rating study (A), word, i.e. *joy*, *surprise* and *sadness* (W) and word dimension, i.e. concreteness, imageability, context availability and valence (D). These factors were hierarchically entered in this order, and the dependent variable was the rating values.

For the word allocation study, the independent variables were participant, i.e. all participants in word allocation study (P), language, i.e. the six languages studied (L), gender, i.e. males and females (G), handedness, i.e. right- and left-handers (H), age, i.e. the ages of the participants in the word allocation study (A), and word, i.e. *joy*, *surprise* and *sadness* (W). These factors were entered in this order for the location values obtained in the X and Y axes; i.e. the two dependent variables in the word allocation study. The variables W, H and L were central to this study and added to the model based on previous research showing that they play a part in the mapping of words onto space (see Marmolejo-Ramos et al., 2013, 2014). Whilst the variable D is specific to the rating task, the variables P and A were peripheral to this study and were included to account for their potential effects on the dependent variables. Some of the estimates of the beta weights of the levels of the independent variables (*β* values) and their associated *t* and *p* values were reported to illustrate their influence on the model. For each hierarchical model, the variability accounted for was estimated as adjusted *R* ^2^ × 100. The models’ fits via ANOVA and robustified *F* tests (*F*_r_).

Avewere comparedrage values and associated measures of deviation were estimated via the median (Mdn) and median absolute deviation (MAD), respectively. The formula $\pm 1.58 \times \left( {\frac{\text{IQR}}{\sqrt n }} \right)$, where IQR = interquartile range and *n* = sample size, was used to generate 95 % CI around the medians for assessing equality of medians at approximately 5 % significance level (see McGill, Tukey, & Larsen, 1978). Based on the results of the robust ANOVA model comparison, pairwise comparisons were examined via the degree of CIs overlap between groups of interest (e.g. within levels of a variable or between variables). Non-overlapping CIs were taken as evidence of significant difference between the groups’ medians (see Cumming, & Finch, 2005; Cumming, 2012). However, when there was some degree of overlap between two or more dependent groups, the Agresti–Pendergast ANOVA test (*F*_AP_) was used via the R function ‘apanova’ (see Wilcox, 2012). The *p* values of multiple comparisons were adjusted via the false discovery rate method, *p*_FDR_ (Benjamini, & Hochberg, 1995). Pairwise comparisons between two or more independent groups were performed via the Cucconi permutation test, MC (Marozzi, 2012, 2014).

## Results

The rating results suggested no differences among the three emotion words regarding their concreteness levels. However, *joy* received higher context availability ratings than *surprise*, and the three words differed in terms of imageability ratings; i.e. *joy* > *surprise* > *sadness*. Central to this study was the finding that, in terms of valence, *joy* was rated higher than *sadness*, and *surprise*’s average ratings fell between the other two words.

### Rating task

Only the models P, P + L + G and P + L + G + H did not have significant *t* and *p* values associated with the *β* values. The other models had significant *β* values [e.g. in the P + L model: *β*_Hindi_ = −1.86 (*t* = −6.65, *p* < 0.001); in the P + L + G + H + A model: *β*_age_ = −0.03 (*t* = −2.88, *p* < 0.01); in the P + L + G + H + A + W model: *β*_sadness_ = −1.78 (*t* = −17.11, *p* < 0.001); and in the P + L + G + H + A + W + D model: *β*_context_ = 1.49 (*t* = 12.42, *p* < 0.001)]. The variability accounted for by each model was 1.02 % (P), 4.57 % (P + L), 4.63 % (P + L + G), 4.66 % (P + L + G + H), 4.82 % (P + L + G + H + A), 10.78 % (P + L + G + H + A + W), and 18.41 % (P + L + G + H + A + W + D). A comparison of the models further suggested that there was an improvement of the fitness of the hierarchical models to the rating data when P, L, and A were added; *F*_r_ = 40.90, *p* < 0.001, *F*_r_ = 22.49, *p* < 0.001 and *F*_r_ = 7.03, *p* = 0.006, respectively. However, the largest improvement occurred when W and D were finally added to the model; *F*_r_ = 111.45, *p* < 0.001 and *F*_r_ = 104.77, *p* < 0.001, respectively.

The model P was significant in that there were differences in the ratings across participants. For example, whereas a participant in the English sample had a median rating of 3.95 [95 % CI (3.15, 4.74)], a participant in the Vietnamese sample had a median rating of 7.7 [95 % CI (4.89, 10.50)]. Language had an effect on the ratings, which was due to median ratings differing across linguistic groups. For example, whilst the median rating in the Hindi sample was 5.4 [95 % CI (5.18, 5.61)], the median rating in the Japanese sample was 6.5 [95 % CI (6.26, 6.73)]. The effect of age on the ratings was graphically explored via a scatter plot with linear and smooth fit lines and a correlation test. The results indicated a near-significant positive correlation (*r*_τ_ = 0.02, *z* = 1.87, *p* = 0.06) such that, for example, the median rating of participants aged 17–25 was 6.7 [95 % CI (6.49, 6.90)], and the median rating of participants aged 30 to 35 was 7.95 [95 % CI (6.70, 9.19)].

The effect of word type (W) was substantiated by the non-overlap between the confidence intervals around the median ratings for the words *joy*, *surprise* and *sadness*; Mdn_joy_ = 7.6 [95 % CI (7.42, 7.77)], Mdn_surprise_ = 6.2 [95 % CI (6.059, 6.34)], and Mdn_sadness_ = 5.8 [95 % CI (5.54, 6.054)].^4^ In the case of the factor word dimension (D), whilst the average ratings in the context and imageability dimensions did not differ {Mdn_context_ = 7.4 [95 % CI (7.20, 7.59)], Mdn_imageability_ = 7.4 [95 % CI (7.24, 7.55)]}, the average ratings in the concreteness and valence dimensions did {Mdn_concreteness_ = 5.7 [95 % CI (5.45, 5.94)], Mdn_valence_ = 5.1 [95 % CI (4.80, 5.39)]}. Also, the ratings for the words in the context and imageability dimensions were higher than the ratings for the words in the concreteness and valence dimensions {Mdn_context+imageability_ = 7.4 [95 % CI (7.27, 7.52)] and Mdn_concreteness+valence_ = 5.2 [95 % CI (5.02, 5.03)]}.

Given the significant effects of W and D on the ratings, their relationship was analysed. Figure 2a shows the ratings of the three words according to the dimension in which they were evaluated. In the concreteness dimension, the median ratings of *joy* {Mdn = 5.7 [95 % CI (5.27, 6.12)]}, *sadness* {Mdn = 5.2 [95 % CI (5.54, 6.25)]} and *surprise* {Mdn = 5.9 [95 % CI (4.73, 5.66)]} did not differ [*F*_AP_ (2, 648) = 1.26, *p* = 0.28]. In the context dimension, there were differences between groups [*F*_AP_ (2, 648) = 4.69, *p* = 0.009] due to the median rating of *joy* {Mdn = 7.6 [95 % CI (7.27, 7.92)]} differing from that of *surprise* {Mdn = 7.2 [95 % CI (6.96, 7.63)]} [*F*_AP_ (1, 324) = 8.68, *p*_FDR_ = 0.01]. Other pairwise comparisons in this dimension, and that involved the word *sadness* {Mdn = 7.3 [95 % CI (6.88, 7.51)]}, were not significant (all *p*_FDR_ > 0.05). There were also differences between *joy* {Mdn = 7.8 [95 % CI (7.58, 8.01)]}, *sadness* {Mdn = 7.5 [95 % CI (7.18, 7.81)]} and *surprise* {Mdn = 7 [95 % CI (6.70, 7.29)]} in the imageability dimension [*F*_AP_ (2, 648) = 14.13, *p* < 0.001] due to all pairwise comparisons being significant (all *p*_FDR_ < 0.05). The non-overlap between the 95 % CIs of *joy* {Mdn = 9.1 [95 % CI (8.89, 9.30)]}, *sadness* {Mdn = 1.65 [95 % CI (1.40, 1.89)]}, and *surprise* {Mdn = 5.1 [95 % CI (4.98, 5.21)]} in the valence dimension indicates that the average ratings between these groups differed significantly.

#### Effects of covariates on the ratings of each emotion word

*Emotion word JOY*: Analyses of the effects of the covariates participant (P), language (L), gender (G), handedness (H), and age (A), on the four types of ratings revealed an effect of P (i.e. P model) on the context availability (CA), imageability (I) and valence (V) ratings of *joy* (CA: *F*_r_ = 15.67, *p* = 5.45*e* ^−05^; I: *F*_r_ = 5.90, *p* = 0.01; V: *F*_r_ = 16.59, *p* = 3.30*e* ^−05^). There was also an effect of L (i.e. P + L model) on the CA and V ratings of *joy* (CA: *F*_r_ = 12.74, *p* = 0.03; V: *F*_r_ = 19.03, *p* = 0.003). All the other models were not significant; *p* > 0.05.

*Emotion word SURPRISE*: Analyses of the effects of the covariates P, L, G, H, and A on the four types of ratings revealed an effect of P on the CA and I ratings of *surprise* (CA: *F*_r_ = 4.16, *p* = 0.03; I: *F*_r_ = 15.58, *p* = 5.74*e* ^−05^). There was also an effect of A (i.e. P + L + G + H + A model) on the V ratings of *surprise* (*F*_r_ = 10.35, *p* = 0.001; a Kendall’s tau test did not support this effect: *τ* = 0.005, *p* = 0.89). All the other models were not significant; *p* > 0.05.

*Emotion word SADNESS*: Analyses of the effects of covariates P, L, G, H, and A on the four types of ratings revealed an effect of P on the concreteness (C), CA, I, and V ratings of *sadness* (C: *F*_r_ = 13.04, *p* < 0.001; CA: *F*_r_ = 29.77, *p* = 2.68*e* ^−08^; I: *F*_r_ = 26.10, *p* = 1.92*e* ^−07^; V: *F*_r_ = 29.96, *p* = 2.43*e* ^−08^). There was also an effect of A (i.e. P + L + G + H + A model) on the C ratings of *surprise* (*F*_r_ = 4.30, *p* = 0.03; *τ* = 0.09, *p* = 0.01), an effect of L (i.e. P + L model) on the CA ratings (*F*_r_ = 18.69, *p* = 0.003), and an effect of G (i.e. P + L + G model) on the I ratings (*F*_r_ = 4.39, *p* = 0.03; a Cucconi test did not support this effect: *MC* = 1.45, *p* = 0.23). All the other models were not significant; *p* > 0.05.

### Word allocation task

The results showed that whilst no one factor had effects on the X-axis data, in the case of the Y axis, regardless of language, gender, handedness and age, *joy* was located in upper spatial locations and *sadness* in lower spatial locations. The neutral emotional concept of *surprise* was located mid-way between joy and sadness. In regard to the language factor, results were in line with those reported by Marmolejo-Ramos et al. (2013) in that there were some differences among linguistic groups in the rating task but none in the word allocation task.

#### Robust linear regression on the X-axis data

In none of the models, the *t* values associated with the *β* values were significant (all *p* > 0.05). The variability accounted for by each model was 0.02 % (P), 0.23 % (P + L), 0.28 % (P + L + G), 0.45 % (P + L + G + H), 0.45 % (P + L + G + H + A), and 0.66 % (P + L + G + H + A + W). A comparison of the models further suggested no improvement of the fitness of the hierarchical models to the *X*-axis data; P model: *F*_r_ = 0.17, *p* = 0.66; P + L model: *F*_r_ = 0.34, *p* = 0.99; P + L + G model: *F*_r_ = 0.44, *p* = 0.49; P + L + G + H model: *F*_r_ = 1.40, *p* = 0.22; P + L + G + H + A model: *F*_r_ = 0.01, *p* = 0.88; and P + L + G + H + A + W model: *F*_r_ = 0.54, *p* = 0.90.

The overlap between the confidence intervals for the words when located in the *X* axis suggests that they are not positioned differently on the horizontal plane (see Fig. 2b). Indeed, although there was variability in the location of the words (MAD_joy_ = 5.93, MAD_surprise_ = 5.93, and MAD_sadness_ = 8.89), the median location for the three words was −1.^5^

#### Effects of covariates on the horizontal position of each emotion word

Analyses of the effects of the covariates participant (P), language (L), gender (G), handedness (H), and age (A) on the X values (e.g. effects of those covariates on the values in the X axis when the word was *joy*) showed that there were non-significant results in the X axis (*p* > 0.05 in all models for each of the three words).

#### Robust linear regression on the Y-axis data

The same analysis described above for the data in the *X* axis was performed for the data in the *Y* axis. Only in the last model, the *t* values associated with the *β* values were significant; e.g. *β*_surprise_ = −2.67 (*t* = −6.66, *p* < 0.001), and *β*_sadness_ = −12.14 (*t* = −29.77, *p* < 0.001). The variability accounted for by each hierarchical model was 0.01 % (P), 0.26 % (P + L), 0.28 % (P + L + G), 0.32 % (P + L + G + H), 0.37 % (P + L + G + H + A), and 49.88 % (P + L + G + H + A + W). A comparison of the models suggested an improvement of the fitness of the hierarchical models to the *Y*axis data only when the predictor W was added; P model: *F*_r_ = 0.19, *p* = 0.66; P + L model: *F*_r_ = 0.40, *p* = 0.99; P + L + G model: *F*_r_ = 0.18, *p* = 0.66; P + L + G + H model: *F*_r_ = 0.29, *p* = 0.58; P + L + G + H + A model: *F*_r_ = 0.46, *p* = 0.49; and P + L + G + H + A + W model: *F*_r_ = 373.43, *p* < 0.001.

The non-overlap between the confidence intervals for the words when located in the *Y* axis suggests that they are positioned differently on the vertical plane (see Fig. 2b). There was some variability in the location of the words (MAD_joy_ = 2.96, MAD_surprise_ = 4.44, and MAD_sadness_ = 4.44), and they had notably different locations on the *Y* axis. Specifically, whilst *joy* was located in the upper end of the square {Mdn_joy_ = 7 [95 % CI (6.46, 7.53)]}, sadness was positioned on the lower end of the square {Mdn_sadness_ = −7 [95 % CI (−7.58, −6.41)]}, and surprise was placed in between the other two words {Mdn_surprise_ = 3 [95 % CI (2.58, 3.41)]}.

#### Effects of covariates on the vertical position of each emotion word

There was an effect of P in the cases of *joy* and *sadness* only (*joy*: P model: *F*_r_ = 2.03, *p* = 0.14; *sadness*: P model: *F*_r_ = 16.46, *p* = 3.54*e* ^−05^), such that some participants allocated these words more upward/downward than others (all other models in *joy* and *sadness* had *p* > 0.05). There was an effect of H in the case of *surprise* only (P + L + G + H model: *F*_r_ = 4.25, *p* = 0.03; a Cucconi test confirmed this difference*: MC* = 3.32, *p* = 0.03), such that right-handers allocated this word higher {Mdn = 3, [95 % CI (2.46, 3.53)]} than left-handers {Mdn = 2, [95 % CI (0.58, 3.41)]}. All the other models in *surprise* had *p* > 0.05 (see Appendix for supplementary results).

## Discussion and conclusions

The aim of the rating task was to characterise the words under scrutiny in their concreteness, context availability, imageability, and valence dimensions. The word allocation task aimed to determine the allocation of these three emotions in space by various linguistic groups. Overall, the results suggest that the valence of the emotion words *joy, surprise* and *sadness* (as indicated on the valence dimension in the rating task) is metaphorically mapped onto the vertical plane, such that *joy* is located in upper locations, *sadness* is located in lower locations and *surprise* is located mid-way between the other two words (word allocation task).

The results of the rating study agree with previous research in which the concreteness, imageability, context availability, and valence of the words *joy*, *sadness* and *surprise* have been assessed (see Table 1; Fig. 2a); however, the present results add novel details. It was found that the three words have similar levels of concreteness and are rated as mildly concrete. Although the results showed that, overall, the three words have medium-to-high levels of imageability, as previous studies have indicated, it was further found that *joy* is more imageable than *sadness*, and *sadness* is more imageable than *surprise*. In addition, the finding that *joy* rated higher than *surprise* in regards to context availability is in line with Marmolejo-Ramos et al. (2014; Tables 1, 2) in which participants generated less emotional contexts for *surprise* than *joy.* The present results thus corroborate the findings of these authors via a rating task. Finally, in agreement with past research, *joy* was rated as more positive than *sadness*, and *surprise* was rated mid-way between the other two emotions. However, the median valence rating of *surprise* {Mdn = 5.1 [95 % CI (4.98, 5.21)]} indicates that this word is regarded as neither positive nor negative. This is a novel finding since it empirically demonstrates that *surprise* is a rather neutral emotion concept. It is interesting to note that we found an effect of language in the rating task, but such a factor did not mediate the word allocation task (see below).

The results of the word allocation study confirm that highly positive emotions such as *joy* are mapped onto upper spatial locations, whilst highly negative emotions such as *sadness* are mapped onto lower spatial locations. This finding is in keeping with research suggesting a metaphorical association between emotion stimuli and the vertical spatial axis (e.g. Ansorge, & Bohner, 2013, Ansorge et al., 2013; Damjanovic, & Santiago, 2016; Marmolejo-Ramos et al., 2014; Meier, & Robinson, 2004; Sasaki et al., 2015, 2016; Xie et al., 2014, 2015). Indeed, the average location of the words on the horizontal axis was no different, and handedness had no effect, which lends extra support to the idea that the vertical plane is more prominent than the horizontal plane for the mapping of emotions onto space as originally suggested by Marmolejo-Ramos et al. (2013). Interestingly, whilst in the rating task, the language and age variables had an influence on the words’ ratings, this was not the case in the word allocation task. As shown in Fig. 1, in the study conducted by Marmolejo-Ramos et al. (2013), the average ratings of words tend to vary across linguistic groups, and as shown by Bird, Franklin and Howard (2001), age of acquisition can correlate with, for instance, the imageability ratings of words. Thus, concluding that language and age have an effect on the ratings of emotion words is not surprising [see for example, Evans, & Levinson (2009) arguments regarding linguistic diversity]. However, in the word allocation task, these factors, along with the factors gender and handedness, did not have any effect. The results of the word allocation task hence suggest that, regardless of language, gender, handedness and age, positive words are located in upper spatial areas and negative words are located in lower spatial areas. This result corroborates the findings from Marmolejo-Ramos et al. (2013).

The novel finding is that *surprise* was located mid-way between *sadness* and *joy* in the vertical axis. Although the median location of *surprise* on the vertical axis was not exactly zero, it was located rather close to it {Mdn = 3 [95 % CI (2.58, 3.41)]}. Numerically speaking, the exact mid-way location in the vertical axis between where *joy* and *sadness* were located is zero, and the exact mid-way location between zero and where *joy* was located is 3.5 (see Fig. 2b). Thus, it could be said that a location above 3.5 should be an indication of the word leaning towards positivity, whilst a value on the *Y* axis below 3.5 should be an indication of the word leaning towards neutrality. Given that the upper arm of the CI around the median rating of *surprise* did not cover 3.5, it is then reasonable to assert that this emotion tends to be located mid-way between *joy* and *sadness* in the vertical spatial plane. This result thus provides further evidence that the neutral emotional valence of *surprise* (as found in the rating task) is reflected in this emotion being mapped mid-way between upper and lower locations onto the vertical plane.

Why is vertical space so salient? It has been argued that locations on the horizontal plane (i.e. left and right) are less salient than locations on the vertical plane (i.e. up and down) since people tend to confuse East–West more than North–South (see Mark, & Frank, 1989, as cited in Marmolejo-Ramos et al., 2013). Locations on the horizontal plane are less noticeable as it is equally easy to look left or right. Locations on the vertical plane, on the other hand, are clear in that locations above eye level are immediately observable and, therefore, more likely to be preferred (i.e. likely to be associated with positive valence) than locations below eye level (see also Freeman, 1975, as cited in Marmolejo-Ramos et al., 2013; see also studies on locatives and comparatives by Clark, Carpenter, & Just, 1973). It is, thus, likely that a mapping of positive-valenced concepts (concepts that refer to events, objects and people) onto upper spatial locations is strongly influenced by bodily configuration and experience rather than language, which labels such experiences.

Note that all studies on the valence–space metaphor focus on mapping of the opposite ends of the affective continuum of a concept (e.g. positive emotions vs negative emotions) onto the opposite ends of the vertical plane (e.g. high spatial location vs low spatial location). The results have consistently shown that high spatial locations are associated with positivity and low spatial locations are associated with negativity (see Clark et al, 1973, and other references cited herein). No previous studies have investigated the location on the vertical plane of neutrally valenced concepts. Our study is the first to show that such concepts, exemplified here with the case of *surprise*, are associated with the mid-point (between *joy* and *sadness*) in the vertical plane.

It is worth noting that focused analyses showed that there were no language effects on the allocation of the three words in the X and Y axes in the first WAT task, but there was a language effect on the allocation of *joy* in the X axis and the allocation of *sadness* in the Y axis in the second WAT task (see Appendix). This finding can be due to simple linguistic variability (see Evans, & Levinson, 2009). Interestingly, no covariate had an effect on the allocation of *surprise* in the vertical and horizontal planes. This suggests that whilst there could be some degree of variability across languages as to the allocation of *joy* and *sadness* in 2D space, there seems to be less variability as to the spatial location of *surprise*. In other words, *surprise* seems to be zeroed in a specific vertical and horizontal coordinate.

This novel result indicates that the location of a concept on the vertical plane mimics the concept’s degree of emotional valence regardless of linguistic background. Indeed, it could be entertained that the location of any stimulus on the vertical plane should mimic the stimulus’ degree of emotional valence. That is, the more positively valenced the stimulus, the higher in vertical space it would be located; likewise, the more negatively valenced the stimulus, the lower it would be located. By the same token, a stimulus that is neither too positive nor too negative would tend to be located towards the middle in the vertical plane, as *surprise* was found to be here. A recent study by Sasaki et al. (2015) could be modified to verify this claim. Sasaki et al. (2015) had participants evaluate emotional images. Before evaluation responses were made, the participants had to swipe the display upward or downward, and then, they made an evaluation of the image’s valence. Surprisingly, when participants swiped upward before the evaluation, a more positive evaluation was given to images, and vice versa. Instead of swiping towards a fixed upper or lower area on the screen, as Sasaki et al. did, participants could be required to freely drag the image along a vertical line which would allow for measurement of the distance from the centre of the screen to the place where the emotional stimulus was dragged to. Then the participants would rate the valence of the stimulus. Based on the current findings, it would be hypothesised that the upper/lower the stimulus is located on the vertical axis on the screen, the more positive/negative it would be rated. This finding would support the claim made by Sasaki et al. (2015) that close temporal associations between somatic information and visual events leads to their retrospective integration and provide further credibility to the findings reported herein.

Whilst the emotions *joy* and *sadness* have distinctive sensorimotor correlates, these correlates are very broad in the case of *surprise*. That is, whilst clapping of hands and head hanging on contracted chest are some of the bodily correlates of *joy* and *sadness*, respectively (see Wallbott, 1998), *surprise* manifests in visual search, eye-brow raising, eye-widening, jaw drop, among others (see Reisenzein et al., 2012). However, given that *surprise* seems to be a neutral emotion, its bodily and sensorimotor correlates can be difficult to pinpoint, and this situation could lead this emotion to not be regarded as an emotion but as a cognitive state (Reisenzein et al., 2012). Given current theories arguing that there are degrees in the embodiment of language and emotions (e.g. Chatterjee, 2010; Marmolejo-Ramos, & Dunn, 2013; Meteyard, Rodríguez, Bahrami, & Vigliocco, 2012), it is possible that as the more neutral a concept (and the object it refers to) becomes, the lower the degree of sensorimotor properties. Such low activation of sensorimotor correlates and neutral valence can be metaphorically mapped onto space in vertical locations that are near the middle instead of upper or lower areas. Moreover, the metaphorical mapping of emotions onto space has so far been limited to the two-dimensional space (i.e. up–down in the *Y* Cartesian coordinate and left–right in the *X* coordinate). It is reasonable to suggest that if valenced concepts were to be allocated in a three-dimensional physical space, highly positively valenced concepts would be placed near the body, highly negatively valenced concepts would be placed far away from the body, and neutrally valenced concepts mid-way between these two. That is, valenced concepts should also have different locations on the *Z* Cartesian coordinate. This is merely conjectural, and further empirical testing is needed to explore this notion.

## Figures and Tables

**Fig. 3:**
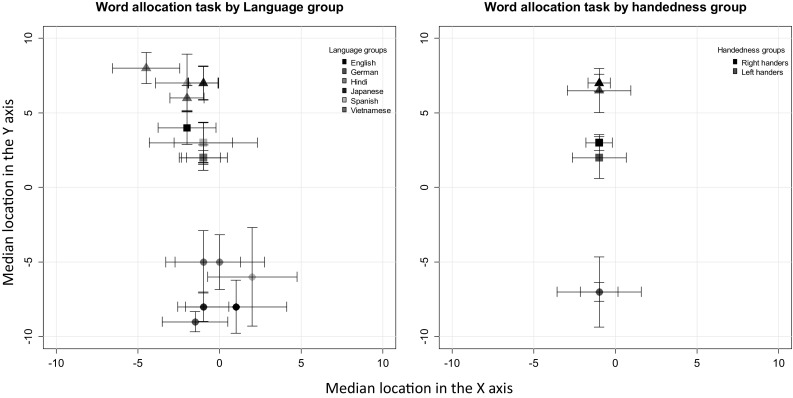
Results of the word allocation task per language and handedness group. The *error bars* represent 95 % CI around the median. *Closed triangle* = joy, *closed square* = surprise and *closed circle* = sadness

## Appendix

### Supplementary graphical results of the non-significant effects of the factors language and handedness in the word allocation task

See Fig. 3.

### Supplementary word allocation task data

Note that in the allocation task reported above, both word order and symbol order were fixed (see Fig. 1). That is, the word order was always joy, surprise and sadness, and they were paired with a triangle, a square and a circle, respectively. Thus, a follow-up study, in which word order (i.e. six possible combinations), symbol order (i.e. also six possible combinations) and their pairings were fully counterbalanced, was conducted (i.e. 36 different word order and symbol order combinations, which gave rise to 36 different paper-based word allocation questionnaires).

A total of 473 participants were randomly allocated to each of the 36 questionnaires (see Table 3). Word order and symbol order were added to the same modelling approach used for the analyses of the data from Study 2. The factors were hierarchically entered in this order: participant (P), language (L), gender (G), handedness (H), age (A), word order (Wo), symbol order (So) and word (W).

**Table 3:** Demographic and descriptive statistic information of the participants in a follow-up study of the word allocation task (MAD = median absolute deviation)

Language	Handedness and gender				Total	Age	
	Right-handed		Left-handed			Range	Median (MAD)
	Male	Female	Male	Female			
+++Study 3 (word allocation task)							
Japanese	43	17	4	1	65	18–35	19 (1.48)
Spanish	93	226	3	16	338	16–57	21 (4.44)
German	18	43	4	5	70	19–48	25 (2.96)
Total	154	286	11	22	473		
Total (handedness)	Right-handers = 440		Left-handers = 33				
Total (gender)	Males = 165		Females = 308				
Total age range						16–57	
Total average age (MAD)							21 (4.44)

The results showed that, as found in Study 2, no factor had a significant effect on the X axis: P model: *F*_r_ = 0.16, *p* = 0.67; P + L model: *F*_r_ = 0.78, *p* = 0.66; P + L + G model: *F*_r_ = 1.75, *p* = 0.17; P + L + G + H model: *F*_r_ = 1.32, *p* = 0.24; P + L + G + H + A model: *F*_r_ = 0.06, *p* = 0.79; P + L + G + H + A + Wo model: *F*_r_ = 0.27, *p* = 0.99; P + L + G + H + A + Wo + So model: *F*_r_ = 0.13, *p* = 0.99; and P + L + G + H + A + Wo + So + W model: *F*_r_ = 5.07, *p* = 0.07. Also, the median X location for the three words was −1: Mdn_joy_ = −1 [95 % CI (−1.36, −0.63)], Mdn_surprise_ = −1 [95 % CI (−1.50, −0.49)], and Mdn_sadness_ = −1 [95 % CI (−1.79, −0.20)].

The analyses also replicated the results in the Y axis shown in Study 2 such that only the model including the factor ‘word’ was significant: P model: *F*_r_ = 0.10, *p* = 0.75; P + L model: *F*_r_ = 0.57, *p* = 0.74; P + L + G model: *F*_r_ = 1.62, *p* = 0.19; P + L + G + H model: *F*_r_ = 0.01, *p* = 0.92; P + L + G + H + A model: *F*_r_ = 1.27, *p* = 0.25; P + L + G + H + A + Wo model: *F*_r_ = 0.37, *p* = 0.99; P + L + G + H + A + Wo + So model: *F*_r_ = 0.86, *p* = 0.97; and P + L + G + H + A + Wo + So + W model: *F*_r_ = 574.37, *p* < 0.001. The median locations for the three words differed: Mdn_joy_ = 7 [95 % CI (6.49, 7.50)], Mdn_surprise_ = 3 [95 % CI (2.56, 3.43)], and Mdn_sadness_ = −7 [95 % CI (−7.72, −6.27)].

Analyses of the effects of the covariates P, L, G, H, A, Wo, and So on the X-axis data for each of the three words showed an effect of L in the allocation of the word *joy* (P + L model: *F*_r_ = 7.58, *p* = 0.01) such that some languages placed this word more rightward/leftward than others (all other models in this word and the words *surprise* and *sadness* had *p* > 0.05). Analyses of the effects of the same covariates on the Y-axis data for each of the three words showed effects of P, L and A in the allocation of the word *sadness* (P model: *F*_r_ = 8.97, *p* = 0.002; P + L model: *F*_r_ = 18.76, *p* = 5.86*e* ^−05^; and P + L + G + H + A model: *F*_r_ = 7.69, *p* = 0.004) such that some participants, languages and age groups allocated this word more upward/downward than others (all other models in this word and the words *surprise* and *joy* had *p* > 0.05).

## Footnotes

1 As discussed at length by other researchers (Niedenthal, Barsalou, Winkielman, Krauth-Gruber, & Ric, 2005), abstract concepts, e.g. emotions, have sensorimotor correlates. Indeed, Holstege (1992) explains how the motor pathways connect to the limbic (i.e. emotion) system. Thus, both abstract and concrete concepts seem to have sensorimotor correlates. There is a two-way interaction between them, which is supported by views of embodied cognition [e.g. Havas, Glenberg & Rinck (2007) show how the activation of sensorimotor systems affect the processing of emotion concepts]. It has to be acknowledged, however, that not all concepts are entirely made up of sensorimotor representations only, and some concepts, e.g. those referring to mental states, can have semantic properties that lack such representations (see Leshinskaya & Caramazza, 2016). It is likely that the activation of non-sensorimotor or sensorimotor properties of a concept is highly task-, stimuli- and context-dependent (evidence in favour of context in concepts’ property activation can be found in Lebois, Wilson-Mendenhall, & Barsalou, 2015).

2 In regards to the concreteness dimension, that emotion words might have medium-to-low levels of concreteness is further confirmed by research showing that the more emotionally laden a word is, the more abstract it is rated (see Kousta, Vigliocco, Vinson, Andrews, & Del Campo, 2011). It is important to note that even if emotion concepts are appended to the category of abstract concepts, there can be an abstract–concrete continuum such that some emotion words are more abstract than others (see chapter 1 in Borghi & Binkofski, 2014). There is a continuum in the abstractness–concreteness spectrum within abstract concepts which mimics the degrees of concreteness (understood as affordances) found in sets of concrete words (see Siakaluk et al., 2008; Xue, Marmolejo-Ramos, & Pei, 2015).

3 It could be argued that the valence–space metaphor could ensue in the horizontal plane when the vertical plane is being controlled for. However, a recent study in which the valence–space metaphor is tested independently in the horizontal and the vertical plane, i.e. one of the planes is being controlled for, showed that such mapping occurs only in the vertical plane (Xie et al., 2015).

4 For clarity, note that that these values are at a group level (not individual level) and are averaged across the four rating dimensions for each word.

5 Even if the medians of the words had aligned towards the left or the right of the square, what matters is that they are aligned; that is, that their median locations in the *X* axis do not differ. If there had been found that, for example, *joy* were around 8, *surprise* were around 0 and *sadness* around −7, then the robust linear modelling should have shown significant effects from any of the variables (e.g. handedness) on the analyses of the *X*-axis data. However, this did not happen.

## References

[CR1] Altarriba J, Bauer LM (2004). The distinctiveness of emotion concepts: A comparison between emotion, abstract, and concrete words. American Journal of Psychology.

[CR2] Altarriba J, Bauer LM, Benvenuto C (1999). Concreteness, context availability, and imageability ratings and word associations for abstract, concrete, and emotion words. Behaviour Research Methods, Instruments and Computers.

[CR3] Ansorge U, Bohner G (2013). Investigating the association between valence and elevation with an implicit association task that requires upward and downward responding. Universitas Psychologica.

[CR4] Ansorge U, Khalid S, König P (2013). Space-valence priming with subliminal and supraliminal words. Frontiers in Psychology.

[CR5] Barsalou LW (2008). Grounded cognition. Annual Review of Psychology.

[CR6] Bekkering H, Neggers SFW (2002). Visual search is modulated by action intentions. Psychological Science.

[CR7] Benjamini Y, Hochberg Y (1995). Controlling the false discovery rate: A practical and powerful approach to multiple testing. Journal of the Royal Statistical Society: Series B.

[CR8] Binder JR, Desai RH (2011). The neurobiology of semantic memory. Trends in Cognitive Sciences.

[CR9] Bird H, Franklin S, Howard D (2001). Age of acquisition and imageability ratings for a large set of words, including verbs and function words. Behavior Research Methods, Instruments, and Computers.

[CR10] Borghi A, Binkofski F (2014). Words as Social Tools: An Embodied View on Abstract Concepts.

[CR11] Bradley, M. M., & Lang, P. J. (1999). Affective norms for English words (ANEW): Stimuli, instruction manual and affective ratings. Technical report C-1, Gainesville, FL. The Center for Research in Psychophysiology, University of Florida.

[CR12] Brysbaert M, Warriner AB, Kuperman V (2014). Concreteness ratings for 40 thousand generally known English word lemmas. Behavior Research Methods.

[CR13] Casasanto D (2009). Embodiment of abstract concepts: Good and bad in right- and left-handers. Journal of Experimental Psychology: General.

[CR14] Casasanto D (2011). Different bodies, different minds: The body specificity of language and thought. Current Directions in Psychological Science.

[CR02] Chatterjee A (2010). Disembodying cognition. Language and Cognition.

[CR15] Clark HH, Carpenter PA, Just MA, Chase WG (1973). On the meeting of semantics and perception. Visual information processing.

[CR16] Cumming G (2012). Understanding the new statistics: Effect sizes, confidence intervals, and meta-analysis.

[CR17] Cumming G, Finch S (2005). Inference by eye. Confidence intervals and how to read pictures of data. American Psychologist.

[CR18] Damjanovic L, Santiago J (2016). Contrasting vertical and horizontal representations of affect in emotional visual search. Psychonomic Bulletin and Review.

[CR19] Evans N, Levinson S (2009). The myth of language universals: Language diversity and its importance for cognitive science. Behavioral and Brain Sciences.

[CR04] Freeman J (1975). The modelling of spatial relations. Computer Graphics and Image Processing.

[CR20] Glenberg AM, Kaschak MP (2002). Grounding language in action. Psychonomic Bulletin and Review.

[CR21] Grühn D, Scheibe S (2008). Age-related differences in valence and arousal ratings of pictures from the International Affective Picture System (IAPS): Do ratings become more extreme with age?. Behavior Research Methods.

[CR22] Hauk O, Pulvermüller F (2011). The lateralization of motor cortex activation to action-words. Frontiers in Human Neuroscience.

[CR23] Havas DA, Glenberg AM, Rinck M (2007). Emotion simulation during language comprehension. Psychonomic Bulletin and Review.

[CR24] Holstege G (1992). The emotional motor system. European Journal of Morphology.

[CR25] Kousta ST, Vigliocco G, Vinson DP, Andrews M, Del Campo E (2011). The representation of abstract words: Why emotion matters. Journal of Experimental Psychology: General.

[CR26] Lebois LA, Wilson-Mendenhall CD, Barsalou LW (2015). Are automatic conceptual cores the gold standard of semantic processing? The context-dependence of spatial meaning in grounded congruency effects. Cognitive Science.

[CR27] Leshinskaya A, Caramazza A (2016). For a cognitive neuroscience of concepts: Moving beyond the grounding issue. Psychonomic Bulletin and Review.

[CR28] Macedo L, Cardoso A, Reisenzein R, Lorini E, Castelfranchi C, Vallverdú J, Casacuberta D (2009). Artificial surprise. Handbook of research on synthetic emotions and sociable robotics: New applications in affective computing and artificial intelligence.

[CR03] Mark, D.M., & Frank, A.U. (1989) Concepts of space and spatial language. In E. Anderson (Ed)., Proceedings of the Ninth International Symposium on Computer-Assisted Cartography (Auto-Carto 9), pp. 538–556, Baltimore, Maryland.

[CR29] Marmolejo-Ramos F, D’Angiulli A (2014). Current research topics in embodied social cognition. Cognitive Processing.

[CR30] Marmolejo-Ramos F, Dunn J (2013). On the activation of sensorimotor systems during the processing of emotionally-laden stimuli. Universitas Psychologica.

[CR31] Marmolejo-Ramos F, Elosúa MR, Yamada Y, Hamm NF, Noguchi K (2013). Appraisal of space words and allocation of emotion words in bodily space. PLoS One.

[CR32] Marmolejo-Ramos F, Montoro PR, Elosúa MR, Contreras MJ, Jiménez-Jiménez WA (2014). The activation of representative emotional verbal contexts interacts with vertical spatial axis. Cognitive Processing.

[CR33] Marocco D, Cangelosi A, Fischer K, Belpaeme T (2010). Grounding action words in the sensorimotor interaction with the world: Experiments with a simulated iCub humanoid robot. Frontiers in Neurorobotics.

[CR34] Marozzi M (2012). A modified Cucconi test for location and scale change alternatives. Revista Colombiana de Estadística.

[CR35] Marozzi M (2014). The multisample Cucconi test. Statistical Methods and Applications.

[CR36] McGill R, Tukey JW, Larsen WA (1978). Variations of box plots. The American Statistician.

[CR37] Meier BP, Robinson MD (2004). Why the sunny side is up: Association between affect and vertical position. Psychological Science.

[CR38] Meteyard L, Rodríguez S, Bahrami B, Vigliocco G (2012). Coming of age: A review of embodiment and the neuroscience of semantics. Cortex.

[CR39] Niedenthal PM, Barsalou LW, Winkielman P, Krauth-Gruber S, Ric F (2005). Embodiment in attitudes, social perception, and emotion. Personality and Social Psychology Review.

[CR40] Paivio A, Yuille JC, Madigan S (1968). Concreteness, imagery, and meaningfulness values for 925 nouns. Journal of Experimental Psychology.

[CR41] Reali F, Arciniegas C (2015). Metaphorical conceptualisation of emotion in Spanish. Two studies on the role of framing. Metaphor and the Social World.

[CR42] Reisenzein R (2009). Emotions as metarepresentational states of mind: Naturalizing the belief–desire theory of emotion. Cognitive Systems Research.

[CR43] Reisenzein R, Meyer W-U, Niepel M, Ramachandran VS (2012). Surprise. Encyclopedia of human behavior.

[CR44] Rueschemeyer S-A, Lindemann O, van Rooj D, van Dam W, Bekkering H (2010). Effects of intentional motor actions on embodied language processing. Experimental Psychology.

[CR45] Sasaki K, Yamada Y, Miura K (2015). Post-determined emotion: Motor action retrospectively modulates emotional valence of visual images. Proceedings of the Royal Society B: Biological Sciences.

[CR46] Sasaki K, Yamada Y, Miura K (2016). Emotion biases voluntary vertical action only with visible cues. Acta Psychologica.

[CR47] Schubert TW (2005). Your highness: Vertical positions as perceptual symbols of power. Journal of Personality and Social Psychology.

[CR48] Schwanenflugel PJ, Shoben EJ (1983). Differential context effects in the comprehension of abstract and concrete verbal materials. Journal of Experimental Psychology, Learning, Memory, and Cognition.

[CR49] Siakaluk PD, Pexman PM, Sears CR, Wilson K, Locheed K, Owen WJ (2008). The benefits of sensorimotor knowledge: Body-object interaction facilitates semantic processing. Cognitive Science.

[CR50] Wallbott HG (1998). Bodily expression of emotion. European Journal of Social Psychology.

[CR01] Wilcox, R. (2012). *Introduction to robust estimation and hypothesis testing*. Amsterdam: Elsevier

[CR51] Wilson M (2002). Six views of embodied cognition. Psychonomic Bulletin and Review.

[CR52] Xie J, Huang Y, Wang R, Liu W (2015). Affective valence facilitates spatial detection on vertical axis: Shorter time strengthens effect. Frontiers in Psychology.

[CR53] Xie J, Wang R, Chang S (2014). The mechanisms of valence-space metaphors: ERP evidence for affective word processing. PLoS One.

[CR54] Xue J, Marmolejo-Ramos F, Pei X (2015). The linguistic context effects on the processing of body-object interaction words: An ERP study on second language learners. Brain Research.

[CR55] Yohai V (1987). High breakdown-point and high efficiency estimates for regression. Annals of Statistics.

